# GPSuc: Global Prediction of Generic and Species-specific Succinylation Sites by aggregating multiple sequence features

**DOI:** 10.1371/journal.pone.0200283

**Published:** 2018-10-12

**Authors:** Md. Mehedi Hasan, Hiroyuki Kurata

**Affiliations:** 1 Department of Bioscience and Bioinformatics, Kyushu Institute of Technology, Kawazu, Iizuka, Fukuoka, Japan; 2 Biomedi Informatics R&D Center, Kyushu Institute of Technology, Kawazu, Iizuka, Fukuoka, Japan; UMR-S1134, INSERM, Université Paris Diderot, INTS, FRANCE

## Abstract

Lysine succinylation is one of the dominant post-translational modification of the protein that contributes to many biological processes including cell cycle, growth and signal transduction pathways. Identification of succinylation sites is an important step for understanding the function of proteins. The complicated sequence patterns of protein succinylation revealed by proteomic studies highlight the necessity of developing effective species-specific *in silico* strategies for global prediction succinylation sites. Here we have developed the generic and nine species-specific succinylation site classifiers through aggregating multiple complementary features. We optimized the consecutive features using the Wilcoxon-rank feature selection scheme. The final feature vectors were trained by a random forest (RF) classifier. With an integration of RF scores *via* logistic regression, the resulting predictor termed GPSuc achieved better performance than other existing generic and species-specific succinylation site predictors. To reveal the mechanism of succinylation and assist hypothesis-driven experimental design, our predictor serves as a valuable resource. To provide a promising performance in large-scale datasets, a web application was developed at http://kurata14.bio.kyutech.ac.jp/GPSuc/.

## Introduction

Different types of protein post-translational modifications (PTMs) serve the proteome with the functional and structural assortment and control cellular dynamics and plasticity [1]. Lysine succinylation is considered one type of PTM, which contributes to regulating many cellular pathology and physiology [2–4]. The succinyllysine was first revealed to occur in the active site of homoserine trans-succinylation processes, while in the intermediate reaction a succinyl assembly was transformed from succinyl-CoA to homoserine [4–7]. Succinylation was found in the regulation of gene transcription [8] and enzyme activities in nucleus, cytoplasm and mitochondria [9–11]. It indicates that lysine succinylation potentially regulates a variety of important biological processes. To identify lysine succinylation, diverse high-throughput proteomic technology has been adopted in numerous organisms by succinylation enrichment and mass spectrometry analyses [3, 6, 7, 10, 12–17]. Nonetheless, improvements in succinylation analysis with experimental identification of protein succinylation sites are still difficult and time-consuming tasks. Owing to various limitations of experimental methods, *in silico* analysis for prediction of succinylation sites is in high demand.

To date, numerous of bioinformatics implementations have been established to predict succinylation substrates [18–27]. Zhao *et al*. proposed a predictor SucPred based on Support Vector Machine (SVM), in which four types of encoding methods were used [18]. The encoding methods include grouped weight based encoding, auto-correlation functions, normalized van der Waals volume and position amino acids weight composition. Another SVM-based predictor iSuc-PseAAC developed by Xu *et al*., adopts the pseudo amino acid composition encoding scheme to improve the prediction performance [19]. Xu *et al*. developed another SVM-based predictor SuccFind considering amino acid composition (AAC), an amino acid index (AAindex) physicochemical properties and k-space amino acid pair composition (CKSAAP) [20]. Jea *et al*. developed two predictors, iSuc-PseOpt [22] and pSuc-Lys [24], by using the general pseudo amino acid composition encoding with random forest (RF) classifiers. Lopez *et al*. developed a structure-based predictor SucStruct using a decision tree classifier [25]. Hasan *et*. *al*. developed two predictors termed as SuccinSite and SuccinSite2.0 based on the amino acid frequency and properties with combined RF classifier scores [21, 23]. The SuccinSite2.0 predictor integrated seven species-specific and their generic model classifiers. This predictor used combination of two sequence features information, i.e. profile-based composition of *k*-spaced amino acid pairs (pCKSAAP) and binary amino acid codes (BE) with a RF classifier. Dehzang et al.http://www.sciencedirect.com/science/article/pii/S0022519317302072 developed two predictors, PSSM-Suc and SSEvol-Suc, based *on* position-specific scoring matrix *(*PSSM) encoding and secondary structure information [27, 28]. Lopez *et al*. developed another predictor, termed Success, using evolutionary and structural properties of amino acids[29]. A specification of those succinylation site prediction tools was summarized in S1 Table.

However, the overall performance of the above-mentioned existing predictors is still not satisfying and there is further room to improve the prediction performance. In the current study, we develop generic and 9 species-specific succinylation classifiers named Global Prediction of Generic and Species-specific Succinylation Sites (GPSuc) based on combining of five sequence encoding features: pCKSAAP, AAC, AAindex, BE, and PSSM features. We optimized the consecutive feature vectors and trained them by a random forest (RF) classifier. With an integration of RF scores *via* logistic regression (LR), the GPSuc outperformed other existing generic and species-specific succinylation site predictors. It provides valuable insights into the processes and functions of succinylation. Moreover, we systematically analyzed critically important features that influence the performance of classifiers. The GPSuc predictor was implemented as a web application at http://kurata14.bio.kyutech.ac.jp/GPSuc/.

## Materials and methods

### Data preparation

One of the main challenges in predicting succinylation sites is to obtain the suitable dataset for model development. Since the training data should be derived from experiments, experimentally identified 10,000 succinylated proteins were collected from nine species. Then the redundant protein samples were removed by using CD-HIT with a 30% identity threshold cutoff [30]. To classify the succinylated proteins, experimentally identified lysine succinylated residues were adopted as positive samples (i.e., succinylation sites), while the remaining lysine residues in these sequences were regarded as negative samples (i.e., non-succinylation sites).

The generic and seven species-specific datasets of *H*. *sapiens*, *M*. *musculus*, *M*. *tuberculosis*, *E*. *coli*, *T*. *gondii*, *S*. *cerevisiae*, and *S*. *lycopersicum* were retrieved from the SuccinSite2.0 [23]. They were the same dataset as the SuccinSite2.0. In a generic model, 124 succinylated proteins with 254 succinylated sites and 2,977 non-succinylated sites were obtained as a test dataset. The training dataset contained 2,198 succinylated proteins with 4,750 validated succinylation and 9,500 putative non-succinylation sites. In addition, after removing 30% sequence redundancy, we collected the datasets of the two species of *T*. *capsulatus* (150 succinylated proteins were set as training samples while 33 proteins randomly as test samples) and *T*. *aestivum* (53 succinylated proteins were set as training samples while 20 proteins randomly as test samples) [15, 16]. It is noted that, in the test dataset, all the succinylation and non-succinylation sites were used and analyzed to simulate the real situation. Training dataset was randomly pooled with a succinylation to non-succinylation site ratio of 1:2. The information of the generic and nine species datasets are listed in S2 Table. The all curated datasets are publicly available at http://kurata14.bio.kyutech.ac.jp/GPSuc/.

### Computational framework

**Fig 1:**
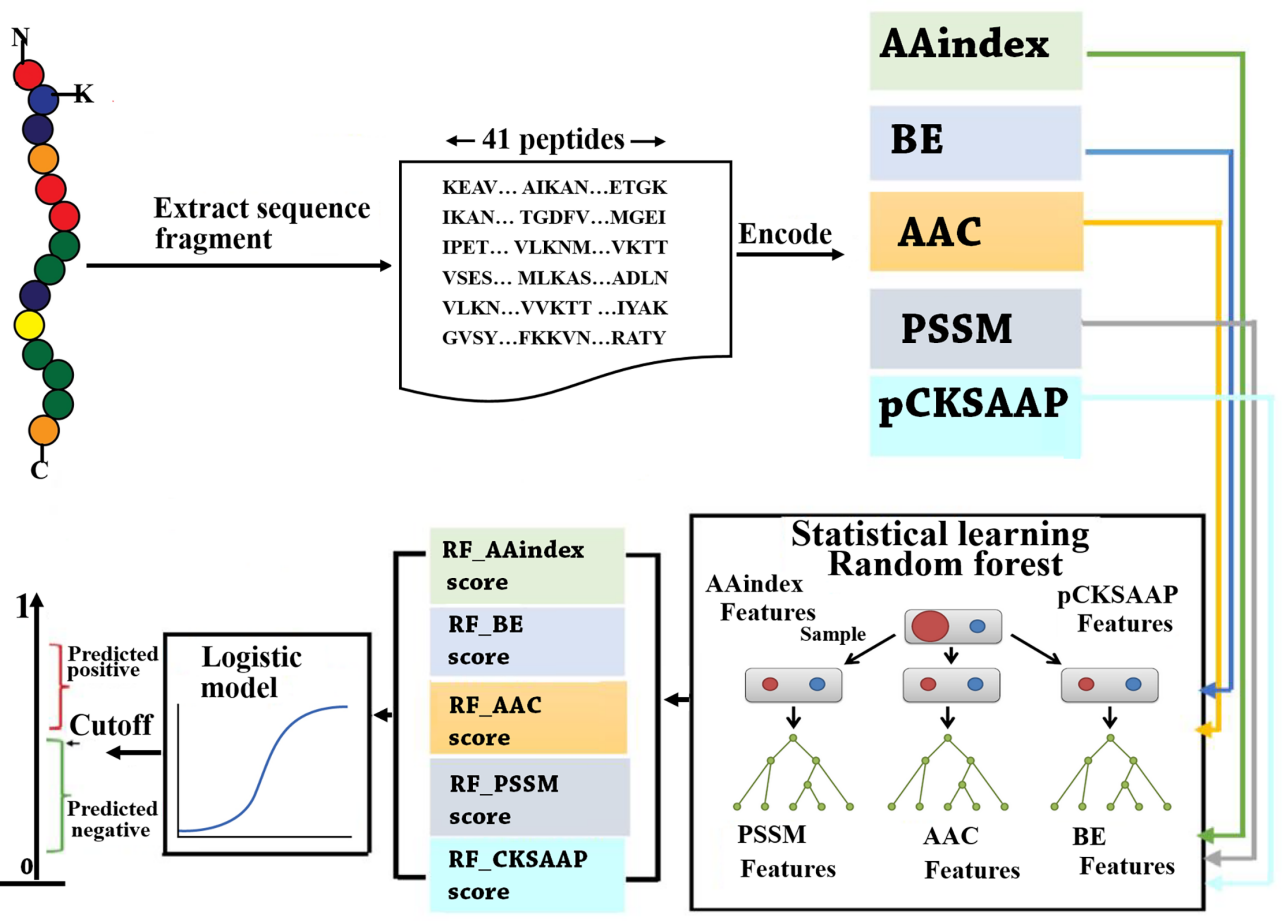
The computational framework of GPSuc.

An overview of the computational framework of the proposed GPSuc predictor is shown in Fig 1. For each of lysine succinylated or non-succinylated proteins, a sequence flanking window of ±20 residues that possesses a succinylated/non-succinylated lysine in the center was considered [23]. When the sequence contains less than 41 amino acids, our method provides gaps (-) to the missing positions to compensate a window size of 41. The sequence window was encoded in the five consecutive features of AAC, BE, AAindex, PSSM, and pCKSAAP. The combination of the feature vectors was optimized using a non-parametric Wilcoxon-rank sum (WR) test. The resulting five collections of the encoded features were independently put into RF models to produce five independent RF prediction scores. Eventually, the five prediction scores by the RF were integrated through the LR method to construct the GPSuc predictor. After combining the prediction scores, a confident cutoff was considered to identify the succinylation site. The optimum RF decision trees were grown up through the training dataset based on the 10-fold CV.

### Features encoding

To establish an accurate species-specific prediction model, the individual sequence fragment was encoded into a numeric feature vector. It is a critical step to represent the collective architecture of the classifier. Therefore, to obtain the local information around each succinylated lysine, a high-quality sequence encoding method was essential. As a substitute for retaining a general binary representation of corresponding amino acid sequences, five types of feature encodings were adopted: AAC, AAindex, BE, PSSM, and pCKSAAP schemes. Details in each feature encoding scheme was described as follows.

### Amino acid composition

AAC feature encoding is one of the most popular schemes and widely used in protein bioinformatics research [26, 31]. It can produce protein sequences information by replicating amino acid occurrence frequencies. In this study, AAC was calculated based on amino acid occurrence frequencies in the sequence fragments surrounding the succinylation and non-succinylation sites (the site itself is not counted). Each of sequence fragments, 20 frequencies were calculated for 20 types of amino acids.

### AAindex encoding

In AAindex database (version 9.1), the primary physicochemical and biochemical properties of the amino acids were extracted [32]. After several trails, twelve types of high-quality amino acid indices such as TSAJ990101[33], MAXF760101 [34], NAKH920108[35], BLAM930101[36], BIOV880101[37], CEDJ970104[38], NOZY710101 [39], KLEP840101[40], NAKH900109 [41], LIFS790101[42], HUTJ700103 (http://www.genome.jp/aaindex/AAindex/list_of_indices) and MIYS990104[43] were transformed into the succinylation and non-succinylation sequence windows for generating the feature vectors. Values “NA” in the amino acid indices were replaced by 0 in this study. In a sequence window through AAindex encoding, a 492-dimension (41×12 = 492) feature vector was generated.

### Binary encoding

A 20-dimensional binary vector for each residue in the sliding window was generated by BE scheme [21]. Through BE, an 820-dimension (41×20 = 820) feature vector was obtained for a sequence fragment.

### PSSM encoding

The PSI-BLAST (version 2.2.26+) against the whole Swiss-Prot non-redundant database (December 2010) was used to generate PSSM matrix [44], which includes two default parameters: e-value cutoff and iteration times. They were set to 1.0×10^−4^ and 3, respectively. Then, the feature vectors were extracted using sliding sequence fragments. To each sequence, the dimension of the PSSM vector was 820 (41×20). We considered 20 amino acids without counting any gap (-).

### pCKSAAP encoding

The compositions of *k*-space amino acid pairs, pCKSAAP feature vectors, were extracted from the generated PSSM profile for each sequence window[45]. If the amino acid residue pair occurs *T* times between *r* and *r+k+1*, the pCKSAAP feature scores were calculated and normalized using the following equation:

$$S_{ij}=\frac{\sum_{i,j=1}^{T}max[\min\left\{PSSM(r,n_{i}),PSSM(r+k+1,n_{j})\right\},0]}{L-k-1}$$ (1)

where *n*_*i*_ and *n*_*j*_ (*i*, *j* = 1, 2, …, 20) represent 20 types of amino acid residues. The PSSM (r, *n*_*i*_) denoted the amino acid pair of *n*_*i*_ with the r^th^ row position of the PSSM score in *n*_*i*_{*k*}*n*_*j*_. The PSSM (r+*k*+1, *n*_*j*_) represents the amino acid pair *n*_*j*_ at the (r+*k*+1)^th^ row position of PSSM. Details in the pCKSAAP scheme are available in our previous study [46]. For each sequence fragment, the dimension of pCKSAAP was 2000 (dimension 5× (20×20) = 2000 at *k* = 0,1,2,3 and 4).

### Wilcoxon rank-sum test

Based on succinylated and non-succinylated samples, five types of features were generated. Among the generated features, there may be some redundant and uncorrelated information, which can affect the speed and accuracy of a predictor. Therefore, feature selection strategies are necessary to pick out informative features that can prevent overfitting, to improve the prediction performance and to understand inherent properties of succinylation sequences. We employed the WR test to select differentially expressed features.

Assuming that a positive-group has *r* scores/observations and a negative-group has *s* scores /observations with test statistics, *W* was defined as the sum of the ranks of the annotations for the positive-group (or negative-group). The following steps were conducted for the WR test.

1. Associate the *r* + *s* annotations with rank observations from the smallest to largest group, where *r* ranks are allocated to the positive-group and *s* ranks are allocated into the negative-group. Calculate *W* of the positive-group.
2. Discover all the possible permutation of the ranks.
3. Each permutation of the rank is calculated and the *p*-value is calculated as follows.

Pupper=(ranksums≤observedranksumofW)/(r+ss) (2)

### Statistical learning

To classify the models of lysine succinylation sites, a supervised statistical learning approach, RF was employed [47]. RF is one of the most precise statistical learning algorithms and provides highly accurate classification results in bioinformatics research [21, 23, 48, 49]. RF works as an ensemble and de-correlated decision trees, which ‘votes’ for one of the two classes, either succinylation or non-succinylation samples. The experimentally verified lysine succinylation samples were labeled ‘+1’, while the other lysine residues labeled ‘-1’. Based on the positive and negative samples, five different types of features were generated using a series of input feature encodings. These generated features were input into RF classifiers to identify whether or not the lysine residues are succinylated.

### Logistic regression

For prediction of succinylated and non-succinylated sites, the outputs of distinct RF scores were combined using an LR method. The LR scheme was successfully used in protein ubiquitin site prediction [50]. The final prediction probability scores were defined:

$$log\left(\frac{P}{1-P}\right)=\sum_{n=1}^{k}\beta_{n}S_{n}+\alpha$$ (3)

where *k* is the number of individual features with probability *P*, *β*_*n*_ is the regression coefficient with prediction score *S*_*n*_ and α is the constant term. A generalized linear model of an R package software (http://www.R-project.org/) was considered to access the LR.

### Performance evaluation

To calculate the prediction performance of each model of GPSuc, the threshold-independent and threshold-dependent indices were measured. The values of area under the curve (AUC) were calculated and the receiver operating characteristic (ROC) curve was depicted using threshold independent parameters by an R-package (https://cran.r-project.org/web/packages/pROC/index.html). Using the threshold dependent parameters, four statistical indexes: accuracy (Ac), specificity (Sp), sensitivity (Sn), and Matthews correlation coefficient (MCC), were calculated, defined as follows:

$$Ac=\frac{nTP+nTN}{nTP+nTN+nFP+nFN}$$ (4)

$$Sp=\frac{nTN}{nTN+nFP}$$ (5)

$$Sn=\frac{nTP}{nTP+nFN}$$ (6)

$$MCC=\frac{nTP\times nTN-nFP\times nFN}{\sqrt{\left(nTN+nFN)\times (nTP+nFP)\times (nTP+nFN)\times (nTN+nFP\right)}}$$ (7)

where *nTP* represents the number of the observed positive residues predicted to be the positive sample, *nTN* the number of the observed negative residues predicted to be the negative sample, *nFP* the number of the observed positive residues predicted to be the negative, and *nFN* the number of the observed negative residues predicted to be the positive sample, respectively.

We used the test dataset to examine the prediction performance of GPSuc. On the other hand, we applied a 10-fold CV test to the training dataset to examine the prediction performance of GPSuc. First, the training dataset was evenly separated into 10 subgroups. One subgroup was given as the test set, and the remaining 9 subgroups as the training set. We repeated this procedure 10 times by changing the training and test samples from 10 subgroups. By calculating the average value of Sp, Sn, Ac, and MCC, the performances of 10-fold results produced a single estimation.

## Results and discussion

### Analysis of compositional biases around succinylation sites

**Fig 2:**
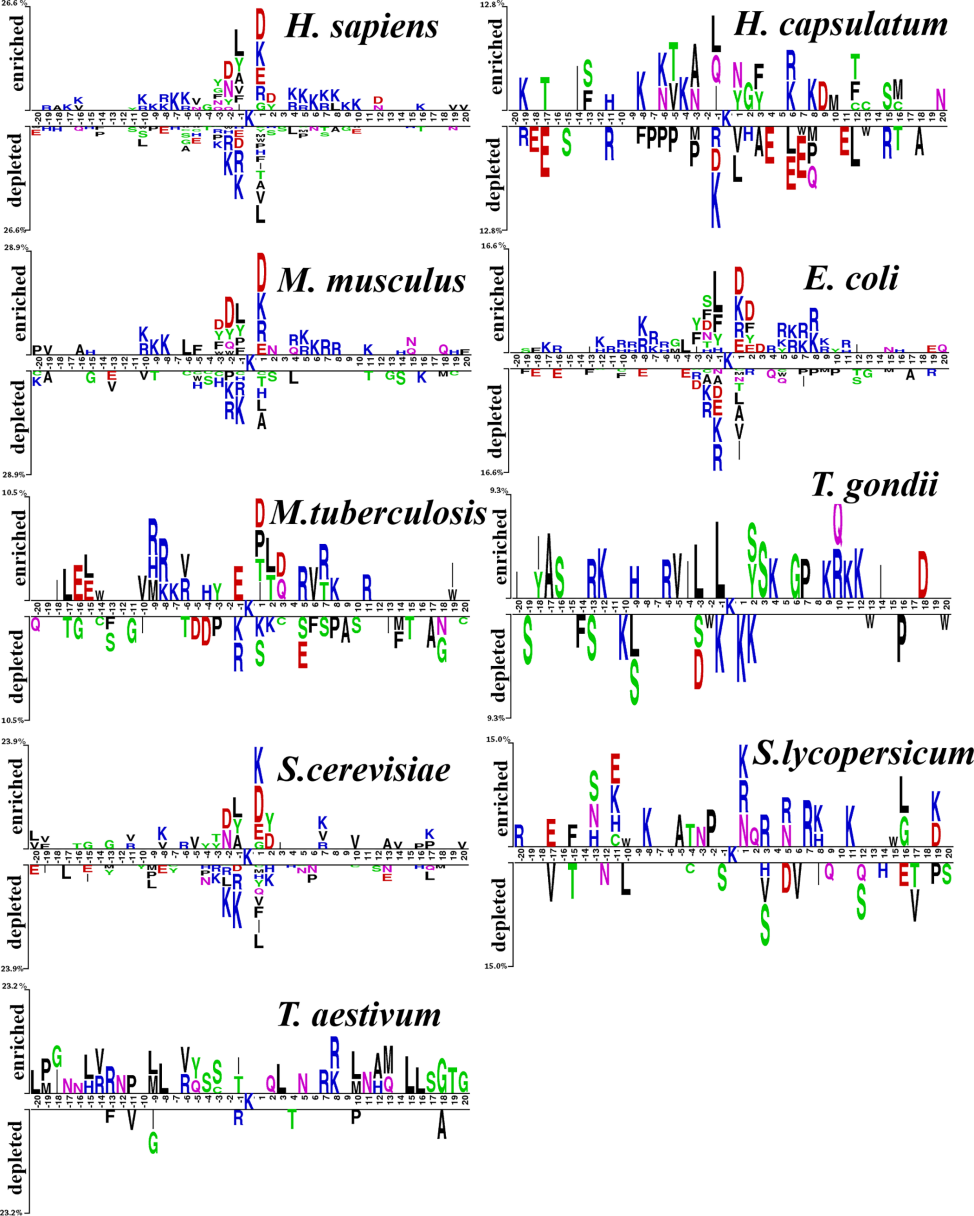
Sequence logos illustrating the amino acid appearance in the sequences surrounding the succinylation sites (http://www.twosamplelogo.org/). Nine species: *H*. *sapiens*, *H*. *capsulatum*, *M*. *musculus*, *E*. *coli*, *M*. *tuberculosis*, *T*. *gondii*, *S*. *cerevisiae*, *S*. *lycopersicum*, and *T*. *aestivum* were used.

First, given that distinct distribution patterns of the sequence surrounding the succinylation sites in the 9 species datasets, a two-sample graph software was used [51] to classify and display the position-specific amino acid appearance in the sequences surrounding the succinylated sites, as shown in Fig 2. In brief, in the two sample logo graphs, the cumulative percentage of over- and under-represented residues was reported with respect to the Y-axis, respectively. Therefore, the letters presented over and under the X-axis indicate frequently observed residues. The sequence patterns for *H*. *sapiens* and *S*. *cerevisiae* resembled each other. Thus, a *H*. *sapiens* succinylation site predictor could be used to predict succinylation sites of *S*. *cerevisiae*. The sequence patterns of succinylation proteins of *H*. *capsulatum*, *M*. *tuberculosis*, *T*. *gondii*, *S*. *lycopersicum*, and *T*. *aestivum* are scattered compared to the other 4 species. For instance, the charged residues (E, K, R and D) were enriched and depleted in *H*. *sapiens*, *M*. *musculus*, *E*. *coli* and *S*. *cerevisiae*. In *M*. *tuberculosis*, *S*. *lycopersicum* and *T*. *aestivum* the neutral residues (C, F, S, and G) were depleted.

**Fig 3:**
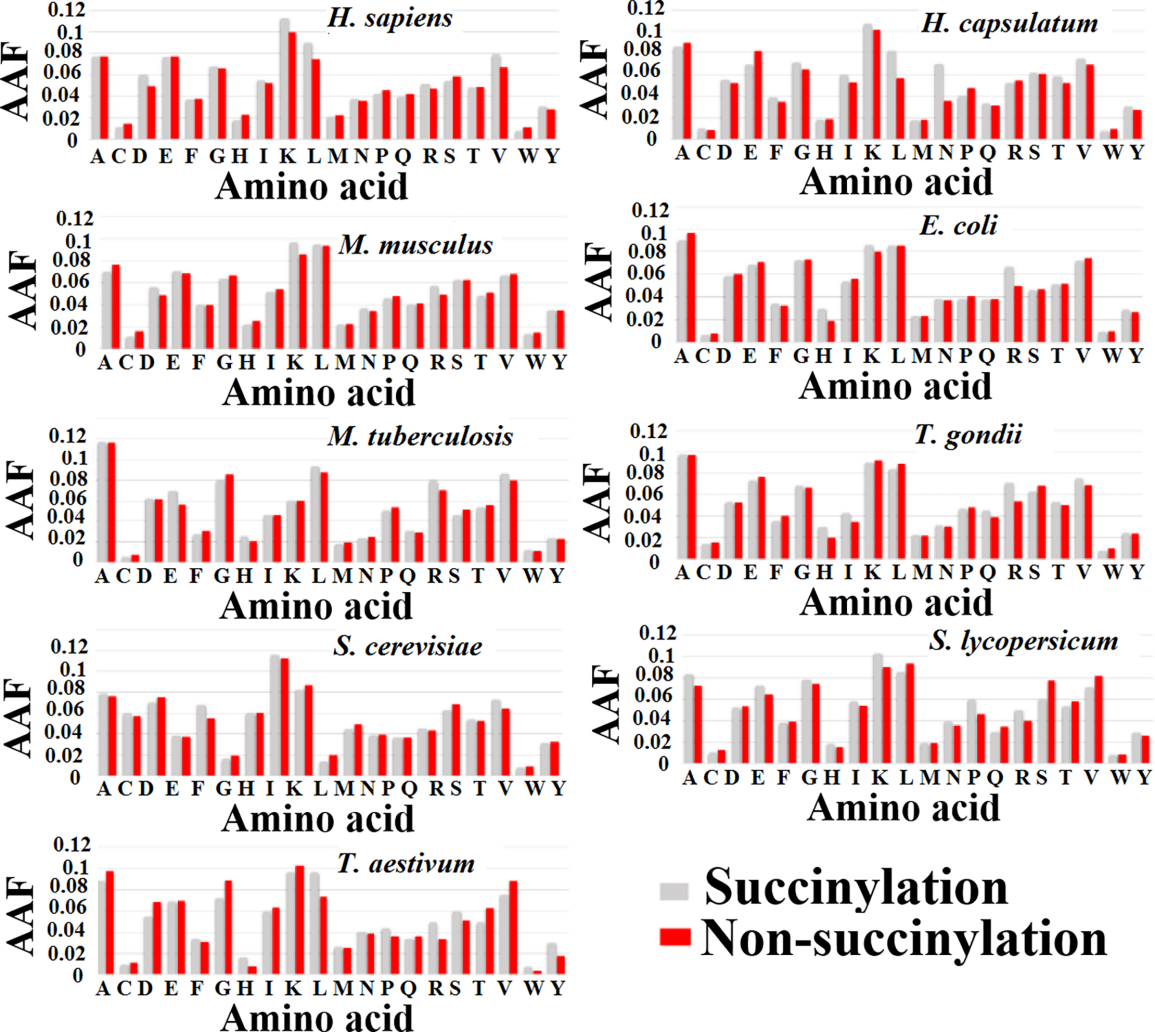
Distribution of AAF in the surrounding succinylation (gray color) and non-succinylation (red color) sequences for nine species. The columns represent AAF, while the rows show each of amino acid residues.

Second, we contemplated the average amino acid occurrence frequency (AAF) scores for each amino acid residue in the surrounding succinylated and non-succinylated sequence windows, as shown in Fig 3. The AAF distribution was found to depend on species. For example, amino acid ‘K’ has very high AAF scores for the 6 species: *H*. *sapiens*, *H*. *capsulatum*, *M*. *musculus*, *S*. *cerevisiae*, *S*. *lycopersicum* and *T*. *aestivum*. Amino acid ‘R’ showed higher AAF scores in *H*. *sapiens*, *E*. *coli*, *M*. *musculus*, *M*. *tuberculosis* and *T*. *aestivum* than the other species. Here, a non-parametric Kruskal-Walis hypothesis test was accessed to identify whether two samples were significantly different. The *p*-values were filtered in the corresponding window positions of neighboring succinylated and non-succinylated sites and corrected by the Bonferroni test. For many amino acids surrounding succinylation sites in the nine species, statistical differences were observed between the succinylated and non-succinylated samples, with a *p*-value of less than 0.05 (S3 Table). These results suggest that the AAF features show visible differences between succinylation and non-succinylation samples in the different species. The AAF could be a useful measure for succinylation site identification.

Third, to detect the distinct amino acids among the succinylated samples in the nine experiential datasets, a chi-square goodness of fit test was conducted. The number of the total succinylated sites were 1405, 382, 438, 760, 2231, 308, 1051, 275 and 145 for nine species of *H*. *sapiens*, *H*. *capsulatum*, *M*. *musculus*, *M*. *tuberculosis*, *E*. *coli*, *T*. *gondii*, *S*. *cerevisiae*, *S*. *lycopersicum* and *T*. *aestivum*, respectively (S2 Table). The amino acid occurrence numbers at different sequence window positions (~-5 to +5) were detected. The statistical differences in the amino acid occurrence numbers between succinylated and non-succinylated samples were calculated by the Bonferroni correction test (S4 Table). We found that most of *p-*values were lower than 0.01, indicating that the amino acid residues of nine species-specific models are significantly different. The above analysis recommended that the lysine succinylation sites across different species have distinctive location-specific modifications. It is, therefore, essential to construct an accurate prediction of species-specific succinylation sites.

### Analysis of evolutionary features of succinylation sites

In the PTM analysis, evolutionary information is an important representative feature [23, 35, 52, 53]. The PSSM feature was considered to measure the evolutionary conservative information around the succinylated and non-succinylated samples. S1 Fig shows the comparison of the mean PSSM values (MPV) between the succinylated and non-succinylated samples for nine species. In *H*. *sapiens*, *H*. *capsulatum*, *E*. *coli*, *S*. *cerevisiae*, *S*. *lycopersicum* and *T*. *aestivum*s species, the MPVs of the surrounding succinylated sites showed higher scores than those of the non-succinylated ones. It suggested that succinylated samples have a tendency to be more conserved than non-succinylated samples. Furthermore, to examine whether succinylated and non-succinylated sites are significantly dissimilar, a non-parametric Kruskal-Walis test was performed. The calculated and filtered *p*-values were adjusted by the Bonferroni test (S5 Table). The MPVs of some window positions of the surrounding succinylated and non-succinylated sites were found significantly different with *p-*value < 0.05, indicating that the PSSM features can capture evolutionary information of the local sequences.

### Analysis of physicochemical properties of succinylation sites

The property of AAindex is the most spontaneous feature in PTM prediction tasks. In the preceding work, different AAindex properties were used [18, 21], which demonstrated that physicochemical properties play a significant role in succinylation site prediction. After several trials, 12 types of important AAindex properties were considered (S6 Table). The average values of physicochemical property ‘amino acid composition of multi-spanning proteins’ (NAKH920108) [35] at each position of the succinylation and non-succinylation samples were defined as mean values of physicochemical properties (MPP). The MPPs depended on the species as shown in S2 Fig. Particularly, the MPPs are varied at window positions of -5, −4, -1, +2, +14 and +16. We used the Kruskal-Walis test to assess statistical significance among the nine species. The filtered *p*-values were corrected by the Bonferroni test. The MPPs of some window positions around the succinylation sites were found significantly different with *p-value*< 0.05 (S7 Table).

### Investigation of feature importance and impact in a generic predictor

As mentioned above, to make a more robust generic predictor, we retrieved the same training and test datasets as collected from the SuccinSite2.0 predictor (Materials and Methods). Initially, to inspect the performance for generic site prediction by ‘GPSuc’, the sequence windows were encoded as numerical feature vectors based on the five consecutive features of AAC, BE, AAindex, PSSM, and pCKSAAP. The calculated feature vectors often have redundant and uncorrelated information that impairs the prediction performance. Therefore, feature selection strategies are essential to reduce the dimensionality and optimize the collective contribution features. The feature vectors were optimized using the WR scheme in this study. The WR scheme reduced the dimensionality of the high dimensional pCKSAAP and AAindex features more than other methods. After several trials in the generic classifier, top 390 and 250 feature vectors were collected from the pCKSAAP and AAindex schemes, respectively. The collected feature vectors were transformed into a new ordered feature based on low to high WR values. The corresponding features were adopted from the other three feature vectors (AAC, BE, and PSSM).

The final five encoding feature vectors for a generic model were trained by the RF classifier. The optimum RF decision trees were grown up through the training dataset based on the 10-fold CV. Then the collected RF scores were combined by the LR method to construct GPSuc. The combination of RF scores of five encodings *via* the LR method provided the highest AUC values of the generic classifier were 0.840 and 0.779 for the training and test datasets of the generic model, respectively (Table 1). As observed, the generic predictor performance indexes of Sp, Sn, Ac, and MCC were 0.903, 0.537, 0.781, and 0.498 for the training dataset, respectively (Table 2). The species-specific predictors of the GPSuc showed high performance. In summary, the performance of the generic and species specific classifier of the GPSuc showed high prediction performance.

**Table 1:** AUC values of different combination of feature scores for training and test dataset in a generic predictor.

Datasets	Predictors	AUC
Training	pCKSAAP + AAindex pCKSAAP + AAindex+ Binary pCKSAAP + AAindex+ Binary +AAC pCKSAAP + AAindex+ Binary +AAC+PSSM (GPSuc)	0.827 0.831 0.834 0.840
Test	pCKSAAP + AAindex pCKSAAP + Binary + AAindex pCKSAAP + Binary + AAindex+PSSM pCKSAAP + Binary + AAindex+PSSM+AAC (GPSuc)	0.752 0.767 0.773 0.779

For combining the features, different LR parameters were added.

**Table 2:** Performance of generic and species-specific succinylation site prediction on the training dataset.

Performances	Sp	Sn	Ac	MCC
Generic	0.903	0.537	0.781	0.498
*H*. *sapiens*	0.903	0.545	0.784	0.524
*H*. *capsulatum*	0.901	0.411	0.738	0.39
*M*. *musculus*	0.890	0.512	0.764	0.429
*E*. *coli*	0.890	0.422	0.734	0.408
*M*. *tuberculosis*	0.890	0.289	0.700	0.201
*S*. *cerevisiae*	0.896	0.655	0.816	0.536
*T*. *gondii*	0.896	0.535	0.776	0.519
*S*. *lycopersicum*	0.897	0.478	0.757	0.447
*T*. *aestivum*	0.887	0.418	0.731	0.406

### Performance comparison to existing generic predictors

We evaluated the predictive performances of different succinylation site prediction tools, including iSuc-PseAAC, iSuc-PseOpt, pSuc-Lys, SuccinSite and SuccinSite2.0, as shown in Table 3. The performance evaluation of different schemes is often difficult because they use different training samples with different ratios of positive to negative datasets and diverse assessment procedures. Since many approaches are not publicly available, including SucPred, SuccFind [26], SucStruct [25], PSSM-Suc [27], SSEvol-Suc[28] and Success[29], these six applications were not employed in this study. To make a fair comparison, a test dataset was collected from the published test dataset of SuccinSite2.0 [23]. As shown in Table 3, the generic classifier of GPSuc improved the performances of other existing predictors in terms of Sn and MCC. The GPSuc showed 4% and 9% higher MCC scores than the SuccinSite2.0 and SuccinSite predictors, and outperformed Suc-PseAAC, iSuc-PseOpt and pSuc-Lys predictors. The prediction results proved that the generic classifier of GPSuc is much more powerful and concise than the other existing predictors.

**Table 3:** Performance of exiting generic tools on the test dataset.

Performances/ prediction schemes	Sp	Sn	Ac	MCC
iSuc-PseAAC	0.887	0.122	0.827	0.013
iSuc-PseOpt	0.758	0.303	0.722	0.038
pSuc-Lys	0.826	0.224	0.779	0.036
SuccinSite	0.882	0.371	0.842	0.199
SuccinSite2.0	0.882	0.454	0.848	0.261
GPSuc	0.883	0.499	0.853	0.296

### Species-specific succinylation site prediction of GPSuc

To evaluate the performance of the species-specific classifiers of GPSuc, the test and training samples of the nine species were collected from the SuccinSite2.0 predictor and recently published articles (Materials and Methods). The proposed nine species-specific classifiers were trained and tested based on the consecutive five sequence features of AAC, BE, AAindex, PSSM, and pCKSAAP. To optimize the model features, a WR feature selection strategy was employed by applying a 10-fold CV test to the training dataset of each species. After several trails, the WR feature selection test was found effective in the pCKSAAP and AAindex schemes compared to other model features vectors. Therefore, the optimal feature vectors were transformed from the pCKSAAP and AAindex schemes for nine species. In *H*. *sapiens* model, the top 260 and 440 feature vectors were collected as optimum features from AAindex and pCKSAAP schemes, respectively. Similarly, from AAindex and pCKSAAP schemes, we collected the top 200 and 340 features for *H*. *capsulatum*, the top 150 and 390 features for *M*. *musculus*, the top 200 and 350 features for *E*. *coli*, the top 240 and 350 features for *M*. *tuberculosis*, the top 220 and 450 features for *S*. *cerevisiae*, the top 150 and 290 features for *T*. *gondii*, the top 250 and 450 features for *S*. *lycopersicum* and the top 120 and 400 features for *T*. *aestivum*, respectively. Based on low to high WR scores, these optimal feature vectors were reconstructed into new well-ordered feature vectors.

Five final feature vectors for each species, including the two optimal feature vectors of pCKSAAP and AAindex, were trained by RF classifiers. The collected RF scores were combined by the LR method to construct the GPSuc. Then we plotted the ROC curves and calculated the AUC.

**Fig 4:**
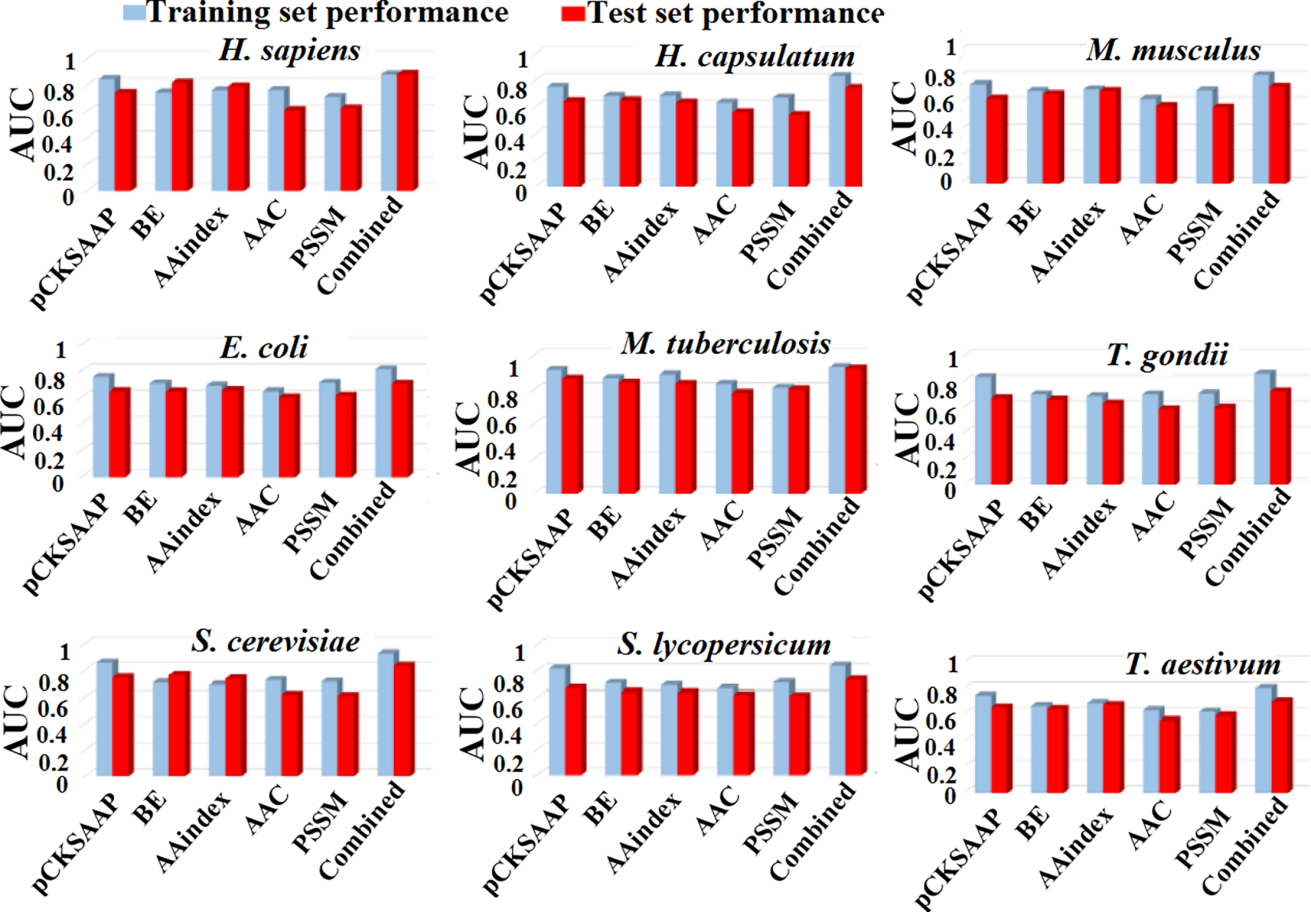
Performance evaluation using single five features and the ‘combined model’ for prediction succinylation sites in nine species. Gray colors represent the AUC value of training dataset while red colors show that of the test dataset. ‘Combined’ indicates the performance by the combined five encoding features. The final *H*. *sapiens* model was given as a linear combination of the five AAC, AAindex, binary, PSSM, and pCKSAAP features with LR coefficient values of 0.142, 1.566, 0.665, 0.342 and 0.667, respectively. In the same way, the combined *H*. *capsulatum*, *M*. *musculus*, *E*. *coli*, *M*. *tuberculosis*, *S*. *cerevisiae*, *T*. *gondii*, *S*. *lycopersicum and T*. *aestivum* were given with (0.102, 0.466, 0.462, 0.242 and 1.367), (0.155, 1.077, 0.575 and 0.761), (0.121, 0.473, 0.763, 0.230 and 1.214), (0.127, 0.358, 0.404, 0.109 and 1.066), (0.320, 0.391, 0.553, 0.182 and 1.122), (0.117, 0.331, 0.734, 0.139 and 1.014), (0.113, 0.417, 0.818, 0.103 and 1.172), and (0.112, 0.462, 0.723, 0.164 and 1.299), respectively. The LR constant terms for each species were set to zero.

**Fig 5:**
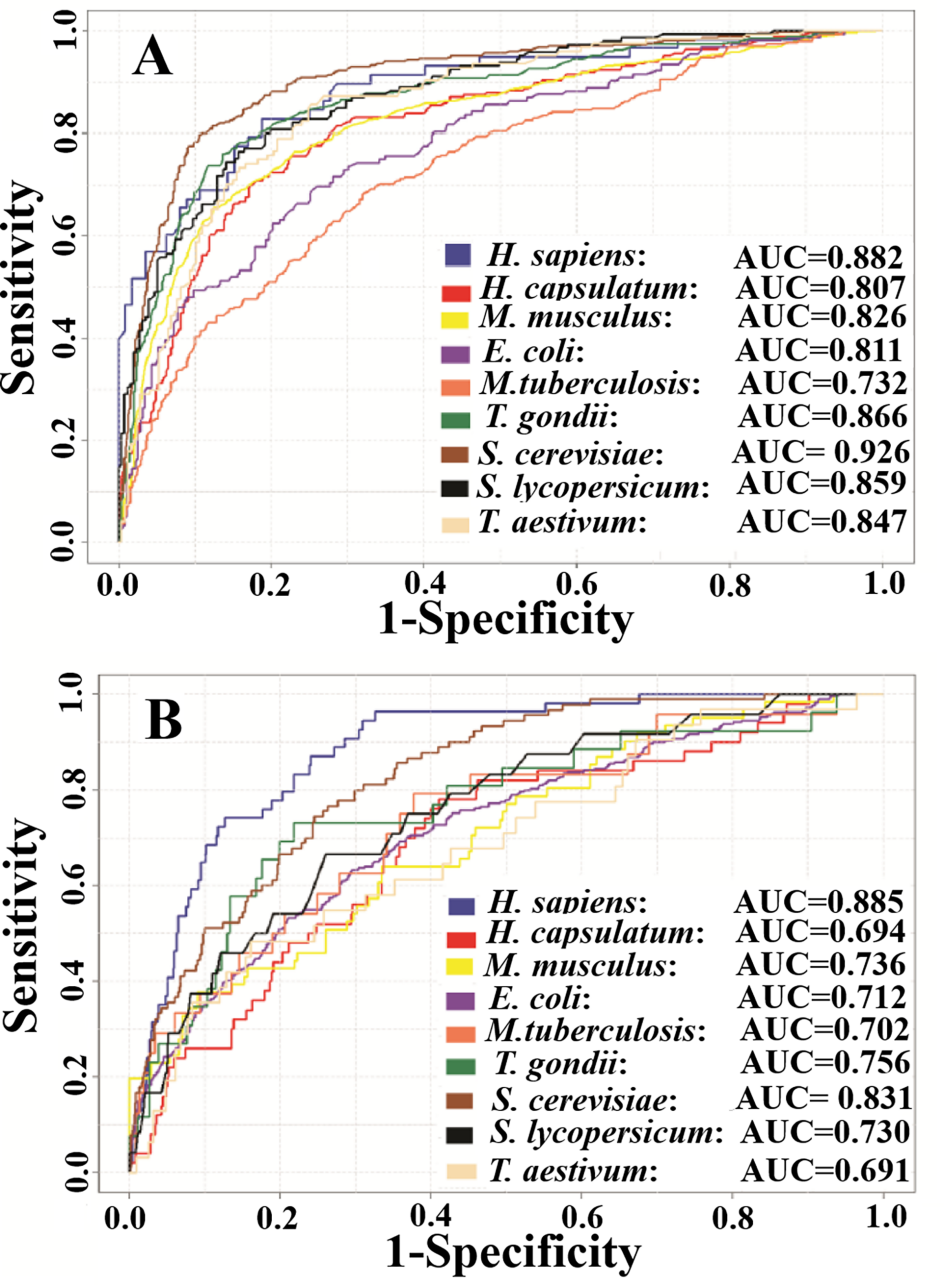
ROC curve of nine species-specific predictors of GPSuc. (A)Training data performances over a 10-fold cross-validation test. (B) Test dataset performances.

The AUC values for each feature encoding model and their combined model (GPSuc) were plotted in a bar plot (Fig 4). The combination of five encoding features *via* the LR method (GPSuc) provided a more powerful predictor than single encoding models. Use of pCKSAAP, AAindex and BE features performed a little higher than the other two features. Using the ROC curves, the performance on the training dataset by the combined model (GPSuc) reached AUC values of 0.882, 0.807, 0.826, 0.811, 0.732, 0.866, 0.926, 0.859 and 0.847 for *H*. *sapiens*, *H*. *capsulatum*, *M*. *musculus*, *E*. *coli*, *M*. *tuberculosis*, *T*. *gondii*, *S*. *cerevisiae*, *S*. *lycopersicum*, and *T*. *aestivum*, respectively (Fig 5A). Finally, the combined models (GPSuc) for the nine species-specific classifiers were evaluated by using test datasets. The GPSuc for the test dataset produced AUC values of 0.885, 0.694, 0.736, 0.712, 0.702, 0.756, 0.831, 0.730 and 0.691 for *H*. *sapiens*, *H*. *capsulatum*, *M*. *musculus*, *E*. *coli*, *M*. *tuberculosis*, *T*. *gondii*, *S*.*cerevisiae*, *S*. *lycopersicum*, and *T*. *aestivum*, respectively (Fig 5B). The above findings support that the proposed species-specific classifiers provide a useful guide to hypothesis-driven experimental design and identification of novel species-specific succinylation sites.

### Comparison with an existing species-specific succinylation site predictor

We compared the performance of the species-specific classifier of GPSuc with SuccinSite2.0, which represents the state-of-the-art predictor available, as shown in Table 4. SuccinSite2.0 is the species-specific classifier for 7 species of *H*. *sapiens*, *M*. *musculus*, *M*. *tuberculosis*, *E*. *coli*, *T*. *gondii*, *S*. *cerevisiae*, *and S*. *lycopersicum* [23]. To make a fair comparison, we employed the same training and test datasets as SuccinSite2.0. The species-specific classifiers of GPSuc for the seven species achieved a much better performance than SuccinSite2.0 in terms of Sn, and MCC (S8 Table). In the test dataset, the above 7 species-specific classifiers of GPSuc provided nearly 4%, 5%, 6%, 5%, 11%, 4% and 5% higher MCCs than the SuccinSite2.0, respectively. In summary, GPSuc outperformed the SuccinSite2.0 predictor.

**Table 4:** Performance comparison of a species-specific predictor using the test dataset.

Species / Measurements	SuccinSite2.0				GPSuc			
	Sp	Sn	Ac	MCC	Sp	Sn	Ac	MCC
*H*. *sapiens*	0.872	0.632	0.866	0.241	0.877	0.693	0.872	0.279
*M*. *musculus*	0.780	0.461	0.769	0.101	0.788	0.523	0.779	0.146
*E*. *coli*	0.733	0.456	0.685	0.192	0.740	0.562	0.710	0.246
*M*. *tuberculosis*	0.720	0.440	0.664	0.139	0.719	0.501	0.675	0.188
*S*. *cerevisiae*	0.826	0.512	0.807	0.216	0.822	0.596	0.809	0.249
*T*. *gondii*	0.824	0.452	0.790	0.191	0.822	0.593	0.801	0.296
*S*. *lycopersicum*	0.815	0.401	0.771	0.172	0.817	0.471	0.800	0.220

## Conclusions

We designed a generic and nine species-specific predictors to classify potential succinylation sites. The GPSuc predictor interpreted high prediction performance in both general and species-specific models. It greatly improved the prediction results compared to previous predictors. Our analysis shows the sequence patterns of succinylation sites are significantly different in the nine species, and the GPSuc combining multiple features using LR analysis improved the prediction performance. To identify the designated succinylation site, a user-friendly online server for GPSuc was established that is particularly beneficial for some hypothesis-driven experiments. GPSuc serves as a complementary and powerful predictor for identification *in vitro* or *in vivo* species-specific succinylation site.

## Supporting information

S1 Table Specification of succinylation site prediction tools. (DOCX)

S2 Table Statistics of the succinylated proteins and the sites of succinylation and non-succinylation in generic and nine species-specific models. (DOCX)

S3 Table Statistical difference in the AAF between the succinylated and non-succinylated samples in nine species. The *p*-values were calculated using the Kruskal-Wallis test and corrected by the Bonferroni multiple comparison test. ‘*’ represents *p* values < 0.05.
(DOCX)

S4 Table Statistical difference in the amino acid occurrence numbers between succinylated and non-succinylated samples at each window position. The *p*-values were calculated using Chi-square and corrected by the Bonferroni test at each window position (~-5 to +5). Nine species of *H*. *sapiens*, *H*. *capsulatum*, *M*. *musculus*, *E*. *coli*, *M*. *tuberculosis*, *S*. *cerevisiae*, *T*. *gondii*, *S*. *lycopersicum* and *T*. *aestivum* are used.
(DOCX)

S5 Table Statistical difference in the MPVs between the succinylated and non-succinylated samples for *H*. *sapiens*, *M*. *musculus*, *E*. *coli*, *M*. *tuberculosis*, *S*. *cerevisiae*, *T*. *gondii*, *and S*. *lycopersicum*. The *p-*values were calculated using the Kruskal Walis test and corrected Bonferroni test. ‘*’ represents *p* values < 0.05.
(DOCX)

S6 Table Twelve types of AAindex properties. They were used for generic and species-specific models.
(DOCX)

S7 Table Statistical difference in the MPP at each position between the succinylated and non-succinylated samples for nine species. The 12 types of AAindex are used to calculate the MPPs for each position of the flanking sequence located in the window positions of ~-20 to +20. The *p* values were calculated using the Kruskal Walis and corrected by Bonferroni test. ‘*’ represents *p* values < 0.05.
(DOCX)

S8 Table Performance comparison of species-specific predictors using the training dataset. (DOCX)

S1 Fig MPV at each position of the succinylated (blue color) and non-succinylated (orange color) samples. The PSSM was used to calculate the MPV at each position of the flanking sequence located in the window positions of ~-20 to +20.
(DOCX)

S2 Fig MPP of the succinylated (blue color) and non-succinylated (orange color) samples for nine species. NAKH920108 was used to calculate the MPP at each position of the flanking sequence located in the window positions of ~-20 to +20.
(DOCX)
