# Supplementary material for: GPSuc: Global Prediction of Generic and Species-specific Succinylation Sites by aggregating multiple sequence features
Source: PLoS One. 2018 Oct 12;13(10):e0200283. doi: 10.1371/journal.pone.0200283 (PMC6193575; doi:10.1371/journal.pone.0200283)
Supplement: S1 Table — (DOCX) [file pone.0200283.s001.docx]

Table S1 Specification of succinylation site prediction tools

| Tools | Species | Sever implementation | Functional server | Statistical learning | Dataset size  (Succinylated proteins/ Sites) | Training(Positive/ Negative) | Testing (Positive/ Negative) | Homolog redundancy | Window size  size | Processing time for a sequence |
| --- | --- | --- | --- | --- | --- | --- | --- | --- | --- | --- |
| SucPred | Generic | http://59.73.198. 144:8088/SucPred/ | No | SVM | 897/2511 | 1436/18,958 | 250/NA | 35% | ~-9 to +9 | N/A |
| iSuc-PseAAC | Generic | http://app.aporc.org/iSuc-PseAAC/ | Yes | SVM | 896/2521 | 1167/3553 | N/A | 40% | ~-15 to +15 | Within 5 sec |
| SuccFind | Generic | http://bio  info.ncu.edu.cn/SuccFind.aspx | No | SVM | 1044/2938 | 2713/23598 | N/A | 30% | ~-10 to +10 | N/A |
| iSuc-PseOpt | Generic | http://www.jci-bioinfo.cn/iSuc-PseOpt | Yes | RF | 896/2521 | 1167/3553 | N/A | 40% | ~-15 to +15 | Within 20 sec |
| pSuc-Lys | Generic | http://www.jci-bioinfo.cn/pSuc-Lys | Yes | RF | 896/2521 | 1167/3553 | N/A | 40% | ~-15 to +15 | Within 20 sec |
| SucStruct | Generic | https://github.com/YosvanyLopez/  SucStruct | No | Decision tree | 670 / 1782 | 1782/1872 | N/A | 40% | ~-15 to +15 | N/A |
| PSSM-Suc | Generic | https://github.com/YosvanyLopez/PSSM-Suc | No | Decision tree | 670 / 1782 | 1782/1643 | N/A | 40% | ~-15 to +15 | N/A |
| SuccinSite | Generic | http://systbio.cau.edu.cn/SuccinSite/ | Yes | RF | 2322/5004 | 4750/9500 | 254/2977 | 30% | ~-13 to +13 | Within 5 sec |
| SuccinSite2.0 | Generic and Species-specific | https://biocomputer.bio.cuhk.edu.hk/SuccinSite2.0/ | Yes | RF | 2322/5004 and Species-specific (see Table S1) | 4750/9500 and Species-specific (see Table S1) | 254/2977 and Species-specific (see Table S1) | 30% | ~-20 to +20 | Within 5 min |

*The N/A indicates that corresponding information of the succinylation predictors does not exist.
