# Supplementary material for: GPSuc: Global Prediction of Generic and Species-specific Succinylation Sites by aggregating multiple sequence features
Source: PLoS One. 2018 Oct 12;13(10):e0200283. doi: 10.1371/journal.pone.0200283 (PMC6193575; doi:10.1371/journal.pone.0200283)
Supplement: S2 Table — (DOCX) [file pone.0200283.s002.docx]

Table S2 Statistics of the succinylated proteins and the sites of succinylation and non-succinylation in generic model and nine species after 30% sequence redundancy.

| Species | Dataset | Succinylated proteins | Succinylation sites | Non-succinylation sites |
| --- | --- | --- | --- | --- |
| Generic | Training | 2,198 | 4,750 | 9,500 |
|  | Test | 124 | 254 | 2,977 |
| *H. sapiens* | Training | 500 | 1,351 | 2,702 |
|  | Test | 50 | 54 | 2,004 |
| *M. musculus* | Training | 240 | 414 | 828 |
|  | Test | 24 | 24 | 679 |
| *E. coli* | Training | 786 | 1,942 | 3,884 |
|  | Test | 79 | 289 | 1,381 |
| *M. tuberculosis* | Training | 369 | 699 | 1,398 |
|  | Test | 36 | 61 | 242 |
| *S. cerevisiae* | Training | 364 | 961 | 1,922 |
|  | Test | 36 | 90 | 1,423 |
| *T. gondii* | Training | 98 | 282 | 564 |
|  | Test | 10 | 26 | 261 |
| *S. lycopersicum* | Training | 150 | 242 | 484 |
|  | Test | 16 | 33 | 274 |
| 1. *capsulatus* | Training | 150 | 332 | 664 |
|  | Test | 33 | 50 | 591 |
| *T. aestivum* | Training | 53 | 113 | 226 |
|  | Test | 20 | 32 | 309 |
