# Supplementary material for: GPSuc: Global Prediction of Generic and Species-specific Succinylation Sites by aggregating multiple sequence features
Source: PLoS One. 2018 Oct 12;13(10):e0200283. doi: 10.1371/journal.pone.0200283 (PMC6193575; doi:10.1371/journal.pone.0200283)
Supplement: S3 Table — The p-values were calculated using the Kruskal-Wallis test and corrected by the Bonferroni multiple comparison test. ‘*’ represents p values < 0.05. (DOCX) [file pone.0200283.s003.docx]

Table S3 Statistical difference in the AAF between the succinylated and non-succinylated samples in nine species. The *p*-values were calculated using the Kruskal-Wallis test and corrected by the Bonferroni multiple comparison test. ‘*’ represents *p* values < 0.05.

| Amino acid | *H. sapiens* | *H. capsulatum* | *M. musculus* | *E. coli* | *M. tuberculosis* | *S. cerevisiae* | *T. gondii* | *S. lycopersicum* | *T. aestivum* |
| --- | --- | --- | --- | --- | --- | --- | --- | --- | --- |
| A  C  D  E  F  G  H  I  K  L  M  N  P  Q  R  S  T  V  W  Y | 1.00  1.00  8.05E-03*  1.00  1.00  7.12E-01  3.91E-01  1.00  4.67E-02*  9.79E-02*  1.00  6.75E-01  1.00  1.00  3.14E-02*  2.37E-01  1.00  4.21E-03*  3.87E-01  4.07E-01 | 3.09E-01  1.00  6.31E-01  4.23E-02*  1.03E-01  9.12E-01  1.00  2.31E-02*  5.13E-02  4.05E-02*  1.00  3.75E-03*  1.00  1.00  1.00  1.00  6.21E-01  3.87E-02*  1.00  1.00 | 4.01E-01  1.00  1.00  2.02E-02*  5.24E-01  1.00  8.91E-01  1.00  4.34E-02*  9.39E-01  1.00  1.00  1.00  1.00  4.31E-02*  1.00  1.00  1.00  1.00  1.00 | 1.00  1.00  2.22E-01  1.00  7.23E-01  2.16E-03*  2.31E-02*  2.19E-02*  3.18E-01  3.02E-04*  1.64E-25*  3.75E-03*  5.73E-01  4.14E-05*  2.48E-03*  1.00  7.21E-03*  1.00  1.00  1.00 | 1.00  1.00  1.00  5.03E-02*  4.43E-01  1.00  1.91E-02*  5.39E-02*  1.00  4.71E-01  1.00  1.00  1.00  1.00  1.37E-02*  1.00  1.00  1.07E-01  1.00  1.00 | 1.00  1.00  4.01E-01  1.00  8.27E-01  6.12E-03*  1.00  1.00  2.17E-02*  2.79E-01  1.64E-25*  1.05E-02*  5.73E-01  1.00  1.00  1.00  1.00  2.37E-02*  1.00  1.00 | 1.00  1.00  3.01E-01  1.00  4.93E-01  1.00  5.03E-03*  2.39E-02*  1.00  1.00  1.00  1.00  1.00  3.10E-01  3.74E-02*  1.00  1.00  2.44E-01  1.00  1.00 | 1.30E-02*  1.00  1.00  1.00  1.00  1.00  1.00  1.00  3.67E-02*  4.79E-01  1.00  6.15E-01  3.39E-03*  3.04E-01  4.21E-02*  2.03E-02*  1.00  3.87E-01  1.00  1.00 | 3.04E-02*  1.00  7.68E-02  1.00  1.00  1.00  1.11E-02*  1.00  1.00  3.07E-03*  1.00  1.00  1.00  1.00  3.35E-02*  8.04E-02  2.01E-01  1.65E-01  1.00  2.43E-02* |
