# Supplementary material for: GPSuc: Global Prediction of Generic and Species-specific Succinylation Sites by aggregating multiple sequence features
Source: PLoS One. 2018 Oct 12;13(10):e0200283. doi: 10.1371/journal.pone.0200283 (PMC6193575; doi:10.1371/journal.pone.0200283)
Supplement: S4 Table — The p-values were calculated using Chi-square and corrected by the Bonferroni test at each window position (~-5 to +5). Nine species of H. sapiens, H. capsulatum, M. musculus, E. coli, M. tuberculosis, S. cerevisiae, T. gondii, S. lycopersicum and T. aestivum are used. (DOCX) [file pone.0200283.s004.docx]

Table S4 Statistical difference in the amino acid occurrence numbers between succinylated and non-succinylated samples at each window position. The *p*-values were calculated using Chi-square and corrected by the Bonferroni test at each window position (~-5 to +5). Nine species of *H. sapiens, H. capsulatum, M. musculus, E. coli, M. tuberculosis, S. cerevisiae, T. gondii, S. lycopersicum* and *T. aestivum* are used.

| Position | A | C | D | E | F | G | H | I | K | L | M | N | P | Q | R | S | T | V | W | Y |
| --- | --- | --- | --- | --- | --- | --- | --- | --- | --- | --- | --- | --- | --- | --- | --- | --- | --- | --- | --- | --- |
| -5 | 1.63E-29 | 9.19E-128 | 5.14E-13 | 1.29E-16 | 3.71E-71 | 7.10E-15 | 1.48E-204 | 2.18E-37 | 1.6E-105 | 3.31E-21 | N/A | 8.71E-22 | 4.09E-08 | 1.32E-17 | 2.62E-71 | 2.71E-61 | 5.01E-17 | 2.01E-73 | 2.08E-115 | 9.43E-36 |
| -4 | 2.23E-52 | 2.42E-176 | 5.31E-53 | 3.28E-17 | 3.39E-25 | 1.19E-39 | N/A | 2.76E-24 | 8.51E-14 | 4.91E-25 | 3.71E-101 | 6.03E-31 | 4.34E-61 | 2.10E-24 | 3.94E-26 | 5.21E-28 | 5.01E-53 | 1.06E-11 | 2.74E-113 | 7.41E-39 |
| -3 | 1.29E-39 | 3.19E-29 | N/A | 2.21E-33 | 4.19E-37 | 5.71E-53 | 3.14E-183 | 6.05E-44 | 3.84E-87 | 8.29E-18 | 6.77E-19 | 1.07E-69 | 5.30E-27 | 3.79E-91 | 3.39E-211 | 2.82E-13 | 2.87E-53 | 4.01E-24 | 1.16E-23 | 4.54E-58 |
| -2 | 3.12E-21 | N/A | 1.81E-05 | 4.65E-68 | 2.15E-77 | 5.05E-37 | 2.12E-23 | 6.65E-54 | 5.12E-104 | 1.13E-39 | 2.17E-107 | 3.46E-217 | 3.69E-62 | 7.85E-15 | 4.48E-26 | 5.31E-18 | 2.41E-69 | 2.41E-53 | 5.04E-15 | 2.41E-16 |
| -1 | 2.24E-37 | 6.28E-28 | 2.28E-176 | 3.46E-58 | 2.78E-16 | 7.06E-36 | 5.27E-205 | 4.35E-102 | 7.42E-66 | 8.14E-39 | 9.15E-28 | 2.26E-208 | 6.58E-177 | 1.86E-14 | 3.03E-176 | 7.57E-07 | 4.02E-93 | 1.50E-55 | 6.13E-256 | 9.87E-26 |
| +1 | 7.73E-33 | 4.11E-203 | 6.91E-101 | 1.19E-13 | 7.49E-3156 | 3.51E-18 | N/A | 5.49E-27 | 7.11E-231 | 2.69E-114 | N/A | 8.04E-27 | 9.31E-109 | 1.18E-31 | 4.23E-88 | 1.84E-59 | 5.03E-68 | 3.56E-19 | N/A | 8.08E-28 |
| +2 | 7.23E-87 | 1.82E-99 | 3.18E-139 | 5.03E-71 | 4.01E-25 | 8.61E-78 | N/A | 8.22E-69 | 7.11E-48 | 4.08E-59 | N/A | 3.02E-39 | 8.11E-14 | 7.81E-16 | 2.37E-17 | 8.87E-42 | 3.58E-29 | 1.46E-18 | 1.72E-16 | 5.83E-158 |
| +3 | 6.03E-03 | 1.04E-252 | 5.01E-123 | 9.81E-12 | 3.21E-75 | 1.04E-49 | N/A | 4.58E-82 | 1.96E-131 | 1.62E-09 | N/A | 2.66E-78 | 4.08E-19 | 1.49E-46 | 1.79E-34 | 3.11E-42 | 2.26E-19 | 4.43E-08 | 615E-107 | 4.47E-41 |
| +4 | 1.39E-23 | 3.15E-71 | 4.29E-03 | 1.25E-22 | 3.21E-18 | 6.43E-39 | 3.57E-27 | 2.84E-48 | 6.30E-135 | 8.81E-09 | 1.81E-54 | 5.40E-41 | 6.14E-22 | 3.17E-21 | 2.48E-41 | 2.19E-18 | 7.19E-15 | 4.43E-09 | 5.01E-29 | 1.65E-156 |
| +5 | 6.19E-16 | 1.69E-11 | 3.94E-38 | 3.53E-17 | 8.21E-42 | N/A | 7.11E-103 | 3.19E-09 | 4.11E-23 | 9.13E-03 | 1.71E-06 | 8.13E-13 | 7.53E-11 | 1.09E-104 | 7.12E-17 | 8.81E-05 | 3.08E-48 | 9.04E-03 | 5.12E-31 | 3.29E-22 |

^a^The N/A indicates that among the nine species at least one corresponding amino acid is missing on the sequence fragments.
