# Supplementary material for: GPSuc: Global Prediction of Generic and Species-specific Succinylation Sites by aggregating multiple sequence features
Source: PLoS One. 2018 Oct 12;13(10):e0200283. doi: 10.1371/journal.pone.0200283 (PMC6193575; doi:10.1371/journal.pone.0200283)
Supplement: S5 Table — The p-values were calculated using the Kruskal Walis test and corrected Bonferroni test. ‘*’ represents p values < 0.05. (DOCX) [file pone.0200283.s005.docx]

Table S5 Statistical difference in the MPVs between the succinylated and non-succinylated samples for *H. sapiens, M. musculus, E. coli, M. tuberculosis, S. cerevisiae, T. gondii, and S. lycopersicum*. The *p-*values were calculated using the Kruskal Walis test and corrected Bonferroni test. ‘*’ represents *p* values < 0.05.

| Window Position | *H. sapiens* | *H. capsulatum* | *M. musculus* | *E. coli* | *M. tuberculosis* | *S. cerevisiae* | *T. gondii* | *S. lycopersicum* | *T. aestivum* |
| --- | --- | --- | --- | --- | --- | --- | --- | --- | --- |
| -20  -19  -18  -17  -16  -15  -14  -13  -12  -11  -10  -9  -8  -7  -6  -5  -4  -3  -2  -1  0  1  2  3  4  5  6  7  8  9  10  11  12  13  14  15  16  17  18  19  20 | 1.00  2.57E-01  1.00  1.00  1.00  2.51E-01  1.00  3.17E-01  1.56E-01  1.24E-01  1.00  6.37E-03*  3.08E-01  1.43E-03*  6.27E-02  1.96E-04*  2.78E-03*  1.00  2.41E-02*  7.18E-03*  2.87E-02*  1.03E-03*  6.54E-03*  1.77E-02*  5.89E-02  2.83E-01  2.56E-02*  6.97E-04*  3.03E-02*  2.75E-04*  3.74E-01  3.81E-03*  1.93E-01  7.23E-03*  2.17E-01  4.98E-02*  8.99E-02*  1.96E-01  1.13E-01  1.71E-01  5.59E-02 | 1.05E-02*  1.29E-03*  5.06E02  6.36E-02  1.42E-02*  1.01E-02*  1.45E-01  5.14E-01  1.56E-01  4.24E-01  1.00  1.05E-01  7.22E-01  1.11E-03*  3.27E-01  1.96E-04*  4.34E-01  1.00  3.41E-02*  4.18E-02*  3.16E-02*  1.31E-02*  9.54E-03*  3.17E-02*  1.07E-02*  1.65E-03*  2.56E-02*  5.17E-02  1.45E-02*  1.37E-02*  2.54E-01  1.01E-03*  3.13E-02  6.51E-04*  2.17E-01  2.35E-02*  6.12E-03*  3.16E-01  2.42E-02*  1.68E-02  1.24E-03* | 1.00  3.06E-02*  1.00  1.00  1.00  1.00  1.00  1.00  1.00  8.25E-01  1.00  1.00  1.00  1.00  1.00  1.00  1.00  1.00  1.00  1.00  4.97E-02*  1.00  1.00  1.00  1.00  1.00  1.00  7.52E-02  4.97E-02*  1.00  1.00  1.00  5.13E-01  1.00  1.00  1.00  1.00  1.00  1.00  1.00  1.00 | 1.00  1.00  1.00  1.00  1.00  8.99E-01  1.00  1.00  1.00  1.00  1.00  1.00  6.57E-03*  1.00  1.00  1.701E-02*  4.17E-02*  3.84E-02*  1.00  1.00  1.00  1.00  6.53E-03*  9.62E-02  7.36E-03*  1.00  2.91E-02*  1.00  6.28E-01  2.93E-01  7.21E-01  6.25E-03*  1.00  1.51E-02*  1.00  4.47E-02*  1.96E-01  1.00  8.21E-01  1.00  1.00 | 1.00  1.00  1.00  1.00  5.91E-03*  7.03E-02  6.52E-01  1.00  1.00  1.00  1.00  6.26E-01  1.79E-02*  1.00  1.00  4.48E-02*  3.36E-02*  1.00  1.65E-02*  1.60E-02*  1.00  1.21E-01  1.00  4.1E-02*  2.62E-02*  3.73E-01  6.00E-01  2.77E-02*  1.00  1.00  1.73E-03*  5.55E-01  5.16E-01  1.00  1.00  1.00  1.00  7.11E-03*  1.00  3.94E-02*  1.00 | 1.00  1.00  3.20E-02*  1.00  1.00  2.41E-02*  1.00  7.24E-01  1.00  1.00  1.00  1.00  1.44E-02*  1.10E-02*  1.38E-03*  1.16E-03*  3.35E-02*  1.00  9.54E-01  1.76E-02*  1.81E-03*  1.00  1.72E-01  2.95E-04*  1.00  1.00  1.00  3.90E-02*  1.00  1.00  5.69E-01  3.20E-02*  9.93E-03*  1.00  2.79E-03*  8.54E-03*  2.42E-01  1.51E-02*  5.99E-04*  3.74E-02*  6.24E-01 | 1.00  1.00  1.00  1.00  1.00  1.00  1.00  1.00  1.00  1.00  1.00  1.00  1.00  1.00  1.00  1.00  1.00  1.00  1.00  1.00  1.00  1.00  1.00  3.04E-02*  1.00E-02*  1.00  1.00  1.00  1.00  1.00  1.00  1.00  6.14E-01  1.00  1.00  1.00  1.00  2.45E-02*  1.00  4.86E-03*  5.25E-02* | 9.91E-03^*^  1.00  1.00  1.00  2.01E-02^*^  1.00  1.00  1.00  1.00  1.00  1.00  1.00  6.30E-02  4.71E-02^*^  1.00  3.34E-02^*^  5.18E-05^*^  4.20E-02^*^  1.00  4.73E-02^*^  1.00  4.65E-01  1.11E-01  3.78E-03^*^  8.50E-01  4.91E-03^*^  1.02E-04^*^  7.85E-02  1.00  1.00  4.01E-02^*^  3.40E-03^*^  9.47E-03^*^  6.53E-03^*^  2.48E-03^*^  4.14E-05^*^  2.64E-02^*^  1.00  5.37E-03^*^  3.88E-03^*^  6.59E-02 | 2.88E-02*  1.00  6.59E-02  1.06E-04*  1.00  1.00  1.00  5.44E-03*  1.00  1.00  4.20E-02*  1.00  2.36E-02*  1.00  1.02E-02*  1.00  1.00  4.95E-02^*^  1.00  1.00  1.63E-02*  3.09E-02*  4.61E-02*  1.08E-04^*^  8.50E-01  4.91E-03^*^  1.02E-04^*^  1.68E-02*  1.00  1.00  1.99E-02^*^  1.00  1.00  3.02E-04^*^  4.04E-01  1.00  1.00  1.45E-02*  4.66E-02^*^  1.36E-02^*^  1.27E-02^*^ |
