# Supplementary material for: GPSuc: Global Prediction of Generic and Species-specific Succinylation Sites by aggregating multiple sequence features
Source: PLoS One. 2018 Oct 12;13(10):e0200283. doi: 10.1371/journal.pone.0200283 (PMC6193575; doi:10.1371/journal.pone.0200283)
Supplement: S6 Table — They were used for generic and species-specific models. (DOCX) [file pone.0200283.s006.docx]

**Table S6**. Twelve types of AAindex properties. They were used for generic and species-specific models.

| AAindex ID | Properties Describtion | Ref |
| --- | --- | --- |
| BLAM930101 | Alpha helix propensity of position 44 in T4 lysozyme | [[1](#_ENREF_1)] |
| MAXF760101 | Alpha and turn propensities | [[2](#_ENREF_2)] |
| TSAJ990101 | Volumes including the crystallographic waters using standard radii and volumes. | [[3](#_ENREF_3)] |
| NAKH920108 | Amino acid composition of MEM of multi-spanning proteins | [[4](#_ENREF_4)] |
| CEDJ970104 | Amino acid composition and cellular location in proteins. | [[5](#_ENREF_5)] |
| LIFS790101 | Conformational preference for all beta-strands | [[6](#_ENREF_6)] |
| NOZY710101 | Transfer energy, organic solvent/water | [[7](#_ENREF_7)] |
| KLEP840101 | Net charge | [[8](#_ENREF_8)] |
| HUTJ700103 | Entropy of formation | (http://www.genome.jp/aaindex/AAindex/list_of_indices) |
| NAKH900109 | Amino acid composition of membrane proteins | [[9](#_ENREF_9)] |
| BIOV880101 | Information value for accessibility | [[10](#_ENREF_10)] |
| MIYS990104 | Optimized relative partition energies | [[11](#_ENREF_11)] |

References

1. Blaber M, Zhang XJ, Matthews BW (1993) Structural basis of amino acid alpha helix propensity. Science 260: 1637-1640.

2. Maxfield FR, Scheraga HA (1976) Status of empirical methods for the prediction of protein backbone topography. Biochemistry 15: 5138-5153.

3. Tsai J, Taylor R, Chothia C, Gerstein M (1999) The packing density in proteins: standard radii and volumes. J Mol Biol 290: 253-266.

4. Nakashima H, Nishikawa K (1992) The amino acid composition is different between the cytoplasmic and extracellular sides in membrane proteins. FEBS Lett 303: 141-146.

5. Cedano J, Aloy P, Perez-Pons JA, Querol E (1997) Relation between amino acid composition and cellular location of proteins. J Mol Biol 266: 594-600.

6. Lifson S, Sander C (1979) Antiparallel and parallel beta-strands differ in amino acid residue preferences. Nature 282: 109-111.

7. Nozaki Y, Tanford C (1971) The solubility of amino acids and two glycine peptides in aqueous ethanol and dioxane solutions. Establishment of a hydrophobicity scale. J Biol Chem 246: 2211-2217.

8. Klein P, Kanehisa M, DeLisi C (1984) Prediction of protein function from sequence properties. Discriminant analysis of a data base. Biochim Biophys Acta 787: 221-226.

9. Nakashima H, Nishikawa K, Ooi T (1990) Distinct character in hydrophobicity of amino acid compositions of mitochondrial proteins. Proteins 8: 173-178.

10. Biou V, Gibrat JF, Levin JM, Robson B, Garnier J (1988) Secondary structure prediction: combination of three different methods. Protein Eng 2: 185-191.

11. Miyazawa S, Jernigan RL (1999) Self-consistent estimation of inter-residue protein contact energies based on an equilibrium mixture approximation of residues. Proteins 34: 49-68.
