# Supplementary material for: GPSuc: Global Prediction of Generic and Species-specific Succinylation Sites by aggregating multiple sequence features
Source: PLoS One. 2018 Oct 12;13(10):e0200283. doi: 10.1371/journal.pone.0200283 (PMC6193575; doi:10.1371/journal.pone.0200283)
Supplement: S7 Table — The 12 types of AAindex are used to calculate the MPPs for each position of the flanking sequence located in the window positions of ~-20 to +20. The p values were calculated using the Kruskal Walis and corrected by Bonferroni test. ‘*’ represents p values < 0.05. (DOCX) [file pone.0200283.s007.docx]

Table S7 Statistical difference in the MPP at each position between the succinylated and non-succinylated samples for nine species. The 12 types of AAindex are used to calculate the MPPs for each position of the flanking sequence located in the window positions of ~-20 to +20. The *p* values were calculated using the Kruskal Walis and corrected by Bonferroni test. ‘*’ represents *p* values < 0.05.

| Window Position | *H. sapiens* | *H. capsulatum* | *M. musculus* | *E. coli* | *M. tuberculosis* | *S. cerevisiae* | *T. gondii* | *S. lycopersicum* | *T. aestivum* |
| --- | --- | --- | --- | --- | --- | --- | --- | --- | --- |
| -20  -19  -18  -17  -16  -15  -14  -13  -12  -11  -10  -9  -8  -7  -6  -5  -4  -3  -2  -1  0  1  2  3  4  5  6  7  8  9  10  11  12  13  14  15  16  17  18  19  20 | 1.00  1.00  1.00  1.00  1.00  1.00  1.00  1.00  1.00  3.22E-01  4.02E-02*  1.00  1.00  1.00  1.00  1.00  1.00  1.00  1.00  2.48E-03*  1.00  4.03E-02*  1.00  1.00  1.00  1.00  1.97E-02*  1.00  1.00  1.00  1.00  3.81E-03*  1.00  1.00  1.00  1.00  1.00  1.00  1.00  1.00  1.00 | 1.00  1.00  1.56E-01  1.00  1.00  1.00  1.63E-02*  1.00  1.56E-01  1.00  1.00  1.00  1.00  1.00  1.00  1.00  1.00  1.00  1.00  4.11E-03*  1.00  2.53E-02*  1.00  1.00  1.00  1.00  4.97E-02*  7.52E-02  1.00  1.00  1.00  1.00  1.00  1.00  2.17E-02*  1.98E-02*  4.99E-02*  1.00  1.00  1.00  1.00 | 1.00  1.00  1.00  1.00  1.00  1.00  1.00  1.00  1.00  1.25E-02*  8.83E-03*  1.00  1.00  1.00  1.00  1.00  1.00  1.00  1.00  1.20E-02*  1.00  1.50E-02*  1.00  1.00  1.00  1.00  1.00  1.00  1.00  1.00  1.00  1.00  5.13E-01  1.00  1.00  1.00  1.00  1.00  1.00  1.00  1.00 | 1.00  1.00  1.00  1.00  1.00  1.00  1.00  1.00  1.00  1.00  1.00  1.00  1.00  1.00  1.00  1.00  1.00  1.00  1.44E-02*  1.00  1.00  3.61E-02^*^  1.00  1.00  1.00  1.00  1.00  1.00  4.71E-01  1.00  1.00  1.00  1.00  1.00  1.00  1.00  1.00  1.00  1.00  1.00  1.00 | 1.00  1.00  1.00  1.00  5.91E-03*  7.03E-02  1.00  1.00  1.00  1.00  1.00  1.00  1.00  1.00  1.00  1.00  1.00  1.00  1.00  1.00  1.00  1.00  1.00  2.01E-02*  1.00  1.00  1.00  1.00  1.00  1.00  1.00  1.00  1.00  1.00  1.00  1.00  1.00  1.00  1.00  1.00  1.00 | 1.00  1.00  1.00  4.76E-02*  1.00  2.06E-02*  1.00  7.24E-01  2.44E-02*  1.00  1.00  1.00  1.00  1.00  1.00  1.00  1.00  1.05E-02*  2.94E-01  1.76E-02*  1.00  1.00  1.00  1.00  1.00  1.00  1.00  1.00  1.00  1.00  1.00  1.00  1.00  1.00  1.00  1.00  1.00  1.00  1.00  1.00  1.00 | 1.00  1.00  1.00  1.00  1.00  1.00  1.00  1.00  1.00  1.00  1.00  1.00  1.00  1.00  1.00  1.62E-02*  1.00  1.00  1.00  1.00E-02*  1.00  3.04E-02*  1.00  1.00  1.0  1.00  1.00  1.00  1.00  1.00  1.00  1.00  1.00  1.00  1.00  1.00  1.00  1.00  1.00  1.00  1.00 | 1.00  1.00  1.00  1.00  1.00  1.00  1.00  1.00  1.00  1.35E-03^*^  3.61E-02^*^  1.00  1.00  1.00  1.00  1.00  1.00  1.00  1.00  1.00  1.00  3.78E-02^*^  1.00  1.00  1.00  1.00  1.00  1.00  1.00  1.00  1.00  1.00  1.00  1.00  1.00  1.0  1.27E-02*  1.00  1.00  1.00  1.00 | 3.1E-02*  1.00  1.23E-02*  1.00  2.01E-02^*^  1.00  1.00  1.00  1.00  1.00  1.00  1.00  2.30E-02^*^  1.00  2.71E-02^*^  1.4E-02^*^  5.18E-01  4.20E-02^*^  1.00  4.73E-02^*^  1.00  4.65E-01  1.11E-01  3.78E-04^*^  250E-01  1.00  1.00  191E-03^*^  1.2E-04^*^  1.00  4.01E-02^*^  1.00  1.00  1.00  1.00  1.53E-02*  1.06E-03*  2.14E-02*  1.00  1.00  1.00 |
