# Supplementary material for: GPSuc: Global Prediction of Generic and Species-specific Succinylation Sites by aggregating multiple sequence features
Source: PLoS One. 2018 Oct 12;13(10):e0200283. doi: 10.1371/journal.pone.0200283 (PMC6193575; doi:10.1371/journal.pone.0200283)
Supplement: S8 Table — (DOCX) [file pone.0200283.s008.docx]

Table S8 Performance comparison of a species-specific predictor using the training dataset

| Species / Performance indexes | SuccinSite2.0 | | | | GPSuc | | | |
| --- | --- | --- | --- | --- | --- | --- | --- | --- |
|  | Sp | Sn | Ac | MCC | Sp | Sn | Ac | MCC |
| *H. sapiens* | 0.891 | 0.491 | 0.758 | 0.486 | 0.903 | 0.545 | 0.784 | 0.524 |
| *M. musculus* | 0.894 | 0.369 | 0.719 | 0.376 | 0.890 | 0.512 | 0.764 | 0.429 |
| *E. coli* | 0.890 | 0.366 | 0.715 | 0.362 | 0.890 | 0.422 | 0.734 | 0.408 |
| *M. tuberculosis* | 0.891 | 0.234 | 0.672 | 0.159 | 0.890 | 0.289 | 0.700 | 0.201 |
| *S. cerevisiae* | 0.896 | 0.632 | 0.808 | 0.501 | 0.896 | 0.655 | 0.816 | 0.536 |
| *T. gondii* | 0.893 | 0.492 | 0.759 | 0.465 | 0.896 | 0.535 | 0.776 | 0.519 |
| *S. lycopersicum* | 0.894 | 0.435 | 0.741 | 0.424 | 0.897 | 0.478 | 0.757 | 0.447 |
