# Supplementary material for: GPSuc: Global Prediction of Generic and Species-specific Succinylation Sites by aggregating multiple sequence features
Source: PLoS One. 2018 Oct 12;13(10):e0200283. doi: 10.1371/journal.pone.0200283 (PMC6193575; doi:10.1371/journal.pone.0200283)
Supplement: S1 Fig — The PSSM was used to calculate the MPV at each position of the flanking sequence located in the window positions of ~-20 to +20. (DOCX) [file pone.0200283.s009.docx]

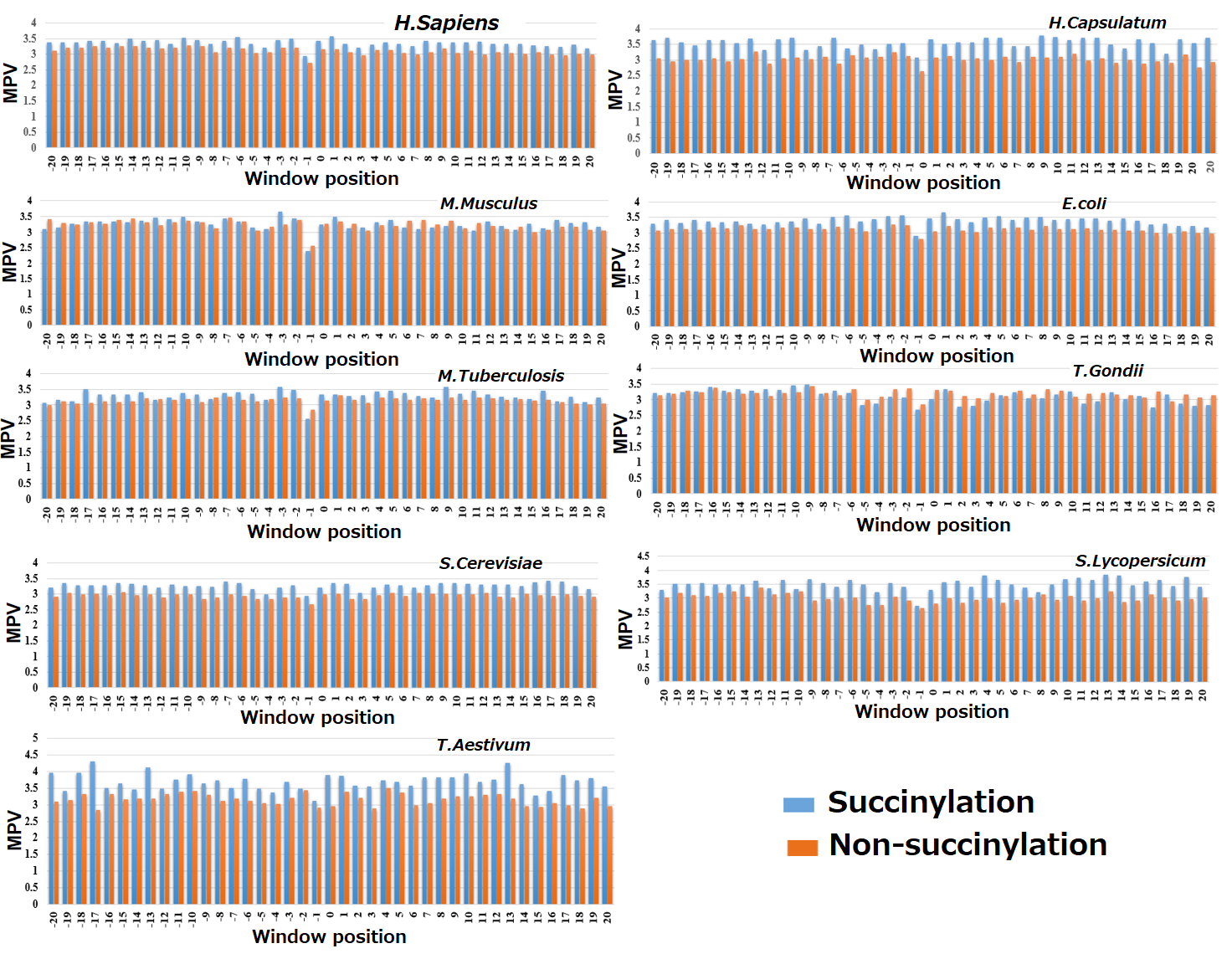


Figure S1 MPV at each position of the succinylated (blue color) and non-succinylated (orange color) samples. The PSSM is used to calculate the MPV at each position of the flanking sequence located in the window positions of ~-20 to +20.
